# Serum and cerebrospinal fluid Neudesin concentration and Neudesin Quotient as potential circulating biomarkers of a primary brain tumor

**DOI:** 10.1186/s12885-019-5525-4

**Published:** 2019-04-05

**Authors:** Olga M. Koper-Lenkiewicz, Joanna Kamińska, Anna Milewska, Karol Sawicki, Marek Jadeszko, Zenon Mariak, Joanna Reszeć, Violetta Dymicka-Piekarska, Joanna Matowicka-Karna

**Affiliations:** 1 Department of Clinical Laboratory Diagnostics, Medical University of Bialystok, ul. Waszyngtona 15A, 15-269 Białystok, Poland; 2 Department of Statistics and Medical Informatics, Medical University of Bialystok, ul. Szpitalna 37, 15-295 Białystok, Poland; 3 Department of Neurosurgery, Medical University of Bialystok, ul. M. Skłodowskiej-Curie 24a, 15-276 Białystok, Poland; 4 Department of Medical Pathomorphology, Medical University of Bialystok, ul. Waszyngtona 13, 15-269 Białystok, Poland

**Keywords:** Biomarker, Cerebrospinal fluid, Neudesin, Primary brain tumor

## Abstract

**Background:**

Despite the previously suggested role of Neudesin in tumorigenesis and its potential as a novel target for the treatment of cancers, its prognostic value has never been examined. Thus, the aim of the study was to evaluate Neudesin concentrations in primary brain tumor patients and make a comparison with non-tumoral individuals.

**Methods:**

Cerebrospinal fluid (CSF) and serum Neudesin concentration was evaluated by means of the ELISA method.

**Results:**

The total group of brain tumor patients had statistically lower serum Neudesin concentrations compared to the non-tumoral group (*P* = 0.037). The meningeal tumor subgroup also had statistically lower serum Neudesin concentrations compared to the non-tumoral group (*P* = 0.012). The Astrocytic brain tumor subgroup had significantly higher CSF Neudesin concentrations compared to the non-tumoral group (*P* = 0.046). Neudesin Quotient (CSF concentration divided by serum concentration) in the astrocytic brain tumor subgroup was statistically higher compared to the non-tumoral group (*P* = 0.023). Males had statistically lower concentrations of the serum Neudesin compared to females (*P* = 0.047). Univariate linear regression analysis revealed that for women the serum Neudesin concentration was 1.53 times higher than for men. In the model of multivariate linear regression analysis, predictor variables influencing serum Neudesin concentrations included CSF Neudesin concentration and the Neudesin Quotient, if other model parameters are fixed. The developed model explains 82% of the variance in serum Neudesin concentration. Both linear regression models, univariate and multivariate, pointed to fewer factors with a potential to influence the Neudesin Quotient compared to serum Neudesin concentration.

**Conclusions:**

In astrocytic brain tumor patients Neudesin concentrations within the cerebrospinal fluid are higher compared with non-tumoral individuals. Serum Neudesin concentration strongly correlates with its CSF level. In primary brain tumor patients serum Neudesin concentration is clearly gender-dependent. Linear regression models pointed to fewer factors that may influence the Neudesin Quotient value, which suggests it is a better biomarker of astrocytic brain tumors than serum and CSF Neudesin concentrations alone.

## Background

The diagnosis and treatment of brain tumors continues to present a significant clinical challenge despite rapid progress in the development of imaging, genomic and proteomic technologies [1]. Their early detection is often hampered by the variability and slow, successive emergence of neurological symptoms [2], as well as a lack of tumor-specific, well defined and easily accessible panels of diagnostic markers [3, 4]. Thus, the quest for circulating diagnostic, prognostic, predictive, and therapeutic response biomarkers of primary brain tumors remains an important objective for modern neurology.

Neudesin (neuron-derived neurotrophic secreted protein) is a secreted protein of 172 amino acids (aa) containing a conserved cytochrome 5-like heme/steroid-binding domain composed of about 100 aa. Although it belongs to the membrane-associated progesterone receptor (MAPR) protein subclass, it is not evolutionarily related to the other members of the same family [5]. The expression of Neudesin is found both in the brain and spinal cord from the embryonic stages to adulthood, as well as in the adipose tissue, lung, heart, and kidney [6, 7]. Neudesin, highly homologous with respect to rodents and humans, has neurotrophic properties and is an active participant in neuronal development and differentiation [8].

Recent studies suggest that Neudesin may be involved in tumorigenesis and tumor invasiveness across the various stages of clinical progression of the disease [8, 9]. Increased expression of Neudesin has been found in tissues of multiple human cancers: malignant lymphoma, breast, uterine cervix, lung, colon, and skin cancers as well as leukemia and breast MCF-7 cell lines [8]. Mechanistic studies revealed that this protein promotes the invasiveness of MCF-7 cells in vitro and increases tumorigenicity in vivo via the activation of MAP (mitogen-activated protein) and PI3K (phosphatidylinositol 3-kinase protein) pathways. *Han* et al. and *Stefanska* et al. found that a loss of Neudesin function promotes cultured cancer cell growth and invasiveness [8, 10].

Despite the previously suggested role of Neudesin in tumorigenesis and its potential as a novel target for the treatment of cancers [8, 10], its prognostic value has never been systematically examined. Thus, the aim of the current study was to retrospectively evaluate Neudesin concentrations in cerebrospinal fluid (CSF) and serum of primary brain tumor patients and compare them to non-tumoral individuals.

In our previous study we revealed significant differences between the astrocytic brain tumor group and non-tumoral individuals in CSF for IL-8, while serum differences were obtained for CCL2 and sICAM-1 (*P* < 0.05). These findings indicate altogether, that for individual biomarkers (IL-8 and CCL2, sICAM-1) the appropriate material, respectively CSF or serum, should be chosen and quantitatively tested [11]. Subsequent searches for brain tumor biomarkers showed that CSF Nogo-A concentrations were lower in patients with CNS tumors compared to non-tumoral subjects (*P* < 0.05). We also found that predictor variables influencing CSF Nogo-A concentrations were diagnosis, sex, and sodium (Na+) level, as the mean CSF Nogo-A concentration in astrocytic brain tumor patients vs. non-tumoral subjects increases for women in comparison to men and changes in relation to sodium levels [12]. Therefore, in order to get a better insight into the cellular transduction pathways involved in the Neudesin effects, we tested possible correlations between Neudesin levels and concentrations of a panel of other cellular factors with previously established functions in brain malignancies: IL-8, CCL2, sICAM-1, and Nogo-A [11, 12]. Further, in order to increase the clinical applicability of our model, we aimed to establish the factors and variables: (e.g.: age, sex, white blood cell count, eGFR value, concentrations of previously tested chemokines, adhesion molecule and inhibitory growth factor) that may influence circulating Neudesin concentration in brain tumor patients.

## Methods

### Subjects

The study population included 28 subjects with previously untreated primary CNS tumors: patients with astrocytic brain tumors and patients with tumors of the meninges (Table 1). The exclusion criterion was a brain tumor remission in medical history.

**Table 1:** Type of tumor, WHO grading and gender of brain tumors patients

Type of tumor (WHO grading)	Gender
Patients with astrocytic brain tumor (11 M/9F; mean age 57 ± years, range 39–73 years)	
Diffuse astrocytoma (2)	M
Glioblastoma (4)	M
Anaplastic astrocytoma (4)	M
Glioblastoma (4)	M
Gliosarcoma (4)	M
Glioblastoma (4)	M
Anaplastic glioma (3)	M
Glioblastoma (4)	M
Pilocytic astrocytoma (1)	M
Glioblastoma (4)	M
Glioblastoma (4)	M
Glioblastoma (4)	F
Diffuse astrocytoma (2)	F
Glioblastoma (4)	F
Glioblastoma (4)	F
Glioblastoma (4)	F
Glioblastoma (4)	F
Glioma (4)	F
Glioblastoma (4)	F
Anaplastic astrocytoma (3)	F
Patients with tumor of the meninges (1 M/7F; mean age 54 ± years, range 36–68 years)	
Anaplastic meningioma (3)	M
Transitional meningioma with psammoma bodies (1)	F
Psammomatous meningioma (1)	F
Transitional meningioma (1)	F
Meningothelial meningioma (1)	F
Fibroblastic meningioma (1)	F
Psammomatous meningioma (1)	F
Meningothelial meningioma (1)	F

*WHO* World Health Organization, *M* male, *F* female

The comparative group was composed of 11 non-tumoral subjects (4 males/7 females; mean age 57 ± years, range 33–70 years) with unruptured intracranial aneurysm, which is usually asymptomatic and discovered incidentally [5]. The exclusion criteria were: cancer in medical history or acute and chronic inflammatory conditions.

Statistical analysis revealed that patient subgroups were age-matched (*P* > 0.05). The laboratory parameters on admission to the hospital of all patients studied were described elsewhere [11].

The study was conducted in agreement with the Helsinki-II-declaration and was approved by the Bioethics Human Research Committee of the Medical University of Bialystok (Permission No. R-I-002/383/2015). All subjects who participated in the study gave their informed written consent.

### Sample collection and storage

CSF specimen collection was performed under a general anesthetic during neurosurgery at the Department of Neurosurgery at the Clinical Medical Hospital in Bialystok as has been described elsewhere [11, 12].

Blood collected in tubes without anticoagulant, in EDTA-K_3_ tubes, and CSF samples were centrifuged for 20 min at 1000 x g. The obtained serum, plasma, and CSF supernatant were stored at − 80 °C until further analysis.

### Neudesin, Nogo-A, IL-8, CCL2, and sICAM-1 concentration analysis

Neudesin, Nogo-A, IL-8, CCL2, and sICAM-1 concentrations were analyzed by means of ELISA (enzyme-linked immunosorbent assay) method, which is an immunological assay. The basis of the test is the binding of the parameter to be determined by specific mono- or polyclonal antibodies bound to the walls of the microplate, acting as a solid phase. The enzyme-labeled antibodies (conjugates) are then applied to the microplate, which bind to the immobilized antigen. The antibodies used have an affinity for various fragments of the parameter under examination, therefore the resulting antibody-antigen complex has a layered arrangement resembling a “sandwich” system. Layering on the substrate plate for the enzyme leads to the colorless solution becoming colored. Stopping the reaction by means of a stop solution leads to a change in color. The color-intensity of the resulting solution is directly proportional to the concentration of the tested antigen in the sample [13].

Neudesin concentration was measured using a BioVendor Human Neudesin ELISA kit (Catalogue No. RD191276200R) in compliance with the manufacturer’s instructions. The detection range for the kit is between 0.06–4.00 ng/mL. CSF and serum were diluted 3-fold prior to analysis.

Nogo-A concentration was measured using a Human RTN4 ELISA kit (Catalogue No. EH3732) from Wuhan Fine Biological Technology Co., Ltd., in compliance with the manufacturer’s instructions. The detection range for the kit is between 78.125–5000 pg/mL. CSF and serum were not diluted prior to analysis.

IL-8 concentration was measured using Quantikine® Human CXCL-8/IL-8 Immunoassay kit (Catalog number: D8000C) from R&D Systems Europe Ltd., Abingdon, England in compliance with the manufacturer’s instructions. The detection range for the kit is between 0.00–2000 pg/mL. CSF and serum were not diluted prior to analysis.

CCL2 concentration was measured using a Quantikine® Human CCL2/CCL2 Immunoassay kit (Catalog number: DCP00) from R&D Systems Europe Ltd., Abingdon, England, in compliance with the manufacturer’s instructions. The detection range for the kit is between 0.00–2000 pg/mL. CSF and serum were diluted 2-fold prior to analysis.

sICAM-1 concentration was measured using a Quantikine® Human ICAM-1/CD54 Allele-specific Immunoassay kit (Catalog number: DCD540) from R&D Systems Europe Ltd., Abingdon, England, in compliance with the manufacturer’s instructions. The detection range for the kit is between 0.00–50 ng/mL CSF and serum were diluted 20-fold prior to analysis.

#### Statistical analysis

The obtained results were statistically analyzed with the use of the STATISTICA 12.0 PL software (StatSoft Inc., Tulsa, USA) and STATA 12.1 (StataCorp LP). The concentrations of the tested parameter did not follow a normal distribution in the preliminary statistical analysis (Shapiro-Wilk test), thus nonparametric statistical analysis was employed. The Mann-Whitney test was used in order to compare two independent samples, and the Kruskal-Wallis test was used for the comparison of three samples. Correlation coefficients were obtained by applying Spearman’s rank method. If not otherwise stated, the values for each given measured variable are stated as median and interquartiles.

We performed a univariate linear regression analysis as well as multiple linear regression analysis to indicate factors that may influence serum/CSF Neudesin concentration and Neudesin Quotient. Tested factors included: diagnosis, age, sex, white blood cell count (WBC), glucose concentration, sodium (Na^+^) and potassium (K^+^) levels, creatinine and eGFR values, as well as previously analyzed proteins: IL-8, CCL2, sICAM-1, and Nog-A concentrations. Significantly skewed variables were logarithmically transformed. When analyzing the results we first built a model for 3 groups (astrocytic brain tumor + meningeal tumor + non-tumoral), however, an analysis of the model assumptions showed a lack of normality of residual distribution, which meant that the model could not be included in the work. Due to the small number of patients in the meningeal group, in the next step of our analysis we built a model for astrocytic brain tumor plus non-tumoral. The separate analysis of patient subgroups is methodologically incorrect. The essence of univariate and multivariate linear regression models is the analysis of the simultaneous impact of the set of factors on the modeled variable. In our models, we analyzed whether there is a statistically significant effect of the diagnosis on the modeled variable. In the case of Neudesin concentration, the effect was statistically insignificant, which means that the parameters of the model do not change significantly for both diagnoses. In the case of Neudesin Quotient, univariate linear analysis showed that the variable statistically significantly differentiates patients due to the diagnosis.

Differences were considered statistically significant for *P* < 0.05.

## Results

Neudesin concentration was analyzed in patients’ serum and cerebrospinal fluid (CSF) samples. Protein concentration was also evaluated in two, randomly selected EDTA-K_3_ plasma samples. The obtained results were as follows: EDTA-K_3_ plasma I: 1.83 ng/mL, serum I: 1.86 ng/mL; EDTA-K_3_ plasma II: 2.16 ng/mL, serum II: 2.17 ng/mL. The limited number of analyzed plasma samples resulted from the available free wells on the ELISA microplate and did not allow for the performing of correlation analysis between values obtained in the plasma with corresponding serum results.

The first step of our analysis was the evaluation of Neudesin concentration in the whole of the brain tumor group (composed of the astrocytic and the meningeal subgroups) to find out if there are differences in Neudesin concentrations in brain tumors in general. Secondly we analyzed Neudesin concentrations separately, depending on the histopathological type of brain tumor, in order to recognize if the levels of the tested protein have a similar or differing trend in individual types of brain tumors.

A similar way of thinking was applied regarding correlation coefficient analysis.

### Serum Neudesin results

The total group of CNS tumor patients had a statistically lower serum Neudesin concentration compared to the non-tumoral group (*P* = 0.037). Individual analysis of tumoral subgroups revealed that the astrocytic brain tumor subgroup as well as the meningeal tumor subgroup had a lower serum Neudesin concentration compared to the non-tumoral group, but a significant difference was found only for the meningeal subgroup vs. the non-tumoral group (*P* = 0.012) (Table 1). Serum Neudesin results did not differ between brain tumor subgroups (astrocytic brain tumor subgroup vs. meningeal tumor subgroup) (*P* > 0.05).

### CSF Neudesin results

The total group of brain tumor patients had a higher CSF Neudesin concentration compared to the non-tumoral group, but it was not statistically significant (*P* > 0.05). The astrocytic brain tumor group also had a higher CSF Neudesin concentration compared to the non-tumoral group and it was statistically significant (*P* = 0.046). On the contrary, the meningeal tumor group showed a lower CSF Neudesin concentration compared to non-tumoral subjects, but it was not significant (*P* > 0.05) (Table 2). CSF Neudesin results did not differ between both CNS tumor subgroups (*P* > 0.05).

**Table 2:** Neudesin concentration [ng/mL] obtained in serum, CSF, and Quotient value of patients with CNS tumors compared to non-tumoral individuals

	Total brain tumor	Astrocytic brain tumor	Meningeal tumor	Non-tumoral group
Serum	1.16 (0.94–1.68)^#^	1.24 (0.92–1.90)	1.07 (0.96–1.37)^#^	1.47 (1.39–1.99)
CSF	1.18 (0.80–2.17)	1.31 (0.93–2.17)^#^	0.66 (0.28–1.79)	0.98 (0.78–1.12)
Quotient	0.98 (0.47–1.33)	0.99 (0.78–1.33)^#^	0.62 (0.28–1.41)	0.56 (0.43–1.00)

^#^statistically significant versus non-tumoral group (*P < 0.05*)

Results are presented as median and interquartiles

### *Neudesin* quotient *results*

To exclude possible impairment of the blood-CSF barrier and/or blood brain barrier (BBB) functions as potential sources influencing the concentration of the tested protein, the CSF concentration was related to the concentration obtained in the serum by calculating the Quotient, as described elsewhere [11, 12, 14, 15]. To calculate the Neudesin Quotient CSF protein concentration was divided by the protein concentration obtained in the serum. Such an approach is well established and minimizes the influence of numerous biological variations (e.g. protein concentration in the blood, patient’s age, CSF flow rate, and the volume of CSF extracted) on the interpretation of Neudesin concentration in the CSF [14, 15]. Moreover, the CSF/serum Neudesin ratio is superior to the CSF total protein, because the interpretation of such a quotient reduces method- and standard-dependent inaccuracy and inter-assay imprecision [14].

The total group of brain tumor patients had increased Neudesin Quotient compared to the non-tumoral group, but it was not significant (*P* > 0.05). Individual analysis of tumoral subgroups revealed that the astrocytic brain tumor subgroup had a statistically higher Neudesin Quotient compared to the non-tumoral group (*P* = 0.023) (Table 2). Neudesin Quotient results did not differ between astrocytic and meningeal tumor subgroups (*P* > 0.05).

### Serum Neudesin results vs. CSF Neudesin results

We did not find statistical differences between Neudesin values obtained in serum compared to CSF concentration, neither in the total group of brain tumors, nor in the astrocytic brain tumor subgroup as well as the meningeal tumor subgroup (*P* > 0.05). Interestingly, the non-tumoral group had a statistically higher serum Neudesin concentration compared to CSF values (*P* < 0.001).

### Male Neudesin results vs. female Neudesin results

In the group of tumoral individuals we found that males had a statistically lower concentration of serum Neudesin (1.01 ng/mL; IQ: 0.91–1.34 ng/mL) compared to females (1.37 ng/mL; IQ: 1.07–2.04 ng/mL) (*P* = 0.047). We did not find any other sex-dependent differences, for either the CSF Neudesin results or the Neudesin Quotient.

In the group of non-tumoral individuals we found that males had a lower concentration of serum Neudesin (1.46 ng/mL; IQ: 1.29–1.67 ng/mL) compared to females (1.94 ng/mL; IQ: 1.39–2.19 ng/mL). We discovered an opposing trend for CSF Neudesin, as males had a higher concentration of protein tested (1.05 ng/mL; IQ: 0.90–1.12 ng/mL) compared to females (0.87 ng/mL; IQ: 0.74–1.24 ng/mL). Males also had a higher Neudesin Quotient (0.64 ng/mL; IQ: 0.58–0.84 ng/mL) compared to females (0.45 ng/mL; IQ: 0.34–1.02 ng/mL). However none of the obtained differences were of statistical significance (*P* > 0.05), which may result from the number of male and female cases under analysis (*N* = 4 and *N* = 7, respectively).

### Correlation coefficients

We tested the correlation coefficient by means of Spearman’s rank method; firstly in the total brain tumor group, secondly in particular patient subgroups, and finally in the non-tumoral group.

In the whole of the brain tumor group (astrocytic plus meningeal tumor) serum Neudesin concentration positively correlated with CSF Neudesin, serum sodium Na^+^ concentration, and age. CSF Neudesin concentration positively correlated with Neudesin Quotient and negatively correlated with eGFR value (Table 3).

**Table 3:** Correlation coefficient results obtained in the whole brain tumors group (astrocytic plus meningeal tumor)

	CSF Neudesin	Neudesin Quotient	Na^+^	eGFR	Age
Serum Neudesin	*R* = 0.696; *P* < 0.001		*R* = 0.503; *P* = 0.006		*R* = 0.419; *P* = 0.026
CSF Neudesin		*R* = 0.747; *P* < 0.001		*R* = −0.517; *P* = 0.023	

In the astrocytic brain tumor subgroup serum Neudesin concentration also correlated positively with their CSF level and serum Na^+^ concentration. CSF Neudesin concentration positively correlated with Neudesin Quotient (Table 4).

**Table 4:** Correlation coefficient results obtained in the group of astrocytic brain tumors

	CSF Neudesin	Neudesin Quotient	Na^+^
Serum Neudesin	*R* = 694; *P* < 0.001		*R* = 0.584; *P* = 0.007
CSF Neudesin		*R* = 0.552; *P* = 0.014	

In the meningeal brain tumor subgroup serum Neudesin concentration showed a strong, positive correlation with CSF Nogo-A levels. CSF Neudesin concentration positively correlated with Neudesin Quotient (Table 5).

**Table 5:** Correlation coefficient results obtained in the group of meningeal tumors

	Neudesin Quotient	CSF Nogo-A
Serum Neudesin		*R* = 0.714; *P* = 0.047
CSF Neudesin	*R* = 0.994; *P* < 0.001	

In the group of non-tumoral individuals serum Neudesin concentration correlated negatively with Neudesin Quotient. CSF Neudesin concentration correlated positively with Neudesin Quotient and negatively with WBC (Table 6).

**Table 6:** Correlation coefficient results obtained in the control group of non-tumoral individuals

	Neudesin Quotient	WBC
Serum Neudesin	*R* = −0.854; *P* < 0.001	
CSF Neudesin	*R* = 0.847; *P* < 0.001	*R* = −0.647; *P* = 0.031

### Univariate and multivariate linear regression analysis results for serum Neudesin

Univariate linear regression analysis results for serum Neudesin revealed that: 1) for women, serum Neudesin concentration is 1.53 times higher than for men; 2) when Neudesin increases in CSF by 1 ng/mL, serum Neudesin concentration increases 1.26 times; 3) with an increase of the Neudesin Quotient by 1.0, the concentration of Neudesin in the serum decreases 1.33 times; 4) with an increase of IL-8 concentration in CSF by 10 pg/mL, the concentration of Neudesin in the serum decreases by 0.5%; 5) with an increase of IL-8 Quotient by 1.0, the concentration of Neudesin in the serum decreases by 0.8%; 6) when the concentration of CCL2 in CSF is increased by 10 pg/mL, the concentration of Neudesin in the serum decreases by 0.2%; 7) when the value of the CCL2 Quotient increases by 1.0, the concentration of Neudesin in the serum decreases by 4%; 8) with an increase in WBC by 1 × 10^3^/μL, serum Neudesin concentration decreases by 3%; 9) with an increase in serum Na^+^ concentration by 10 mmol/L, serum Neudesin concentration increases by 1.6 times (Table 7).

**Table 7:** Univariate and multivariate linear regression analysis results for logarithm of serum Neudesin

No	Covariate	β	e^β^ (95% CI)	*P*-value
		Univariate linear regression analysis		
1	Sex	0.43	1.53 (1.18–1.98)	0.002
2	CSF Neudesin [ng/mL]	0.23	1.26 (1.03–1.54)	0.028
3	Neudesin Quotient	−0.28	0.75 (0.57–0.98)	0.041
4	CSF IL-8 [pg/mL]	−0.0005	0.9995 (0.9991–0.9998)	0.005
5	IL-8 Quotient	−0.0084	0.9917 (0.9856–0.9978)	0.009
6	CSF CCL2 [pg/mL]	−0.0002	0.9998 (0.9996–0.9999)	0.029
7	CCL2 Quotient	−0.04	0.96 (0.93–0.99)	0.012
8	WBC [×10^3^/μL]	−0.03	0.97 (0.94–0.99)	0.036
9	Na^+^ [mmol/L]	0.05	1.05 (1.02–1.08)	0.005
		Multivariate linear regression analysis		
1	CSF Neudesin [ng/mL]	0.62	1.85 (1.64–2.09)	< 0.001
2	Neudesin Quotient	−0.80	0.45 (0.38–0.52)	< 0.001

*CI* Confidence Interval, *CSF* cerebrospinal fluid, *CCL2* Monocyte Chemotactic Protein *1, IL-8* interleukin 8, *Na*^+^ sodium, *WBC* white blood cells count

In the model of multivariate linear regression analysis predictor variables influencing serum Neudesin concentration included: CSF Neudesin concentration and Neudesin Quotient. Adjusted R square (R^2^) for the created model equals 0.82, which indicates that this model explains 82% of the variance in dependent variable.

Adjusted R^2^ shows how well data points fit the curve or line, meaning how much variance has been explained by a model, but adjusting for the number of terms in a model. Adjusted R^2^ calculates R square only from those variables which are significant according to its formulas. Therefore when we are going through a multivariate linear regression we should look at adjusted R^2^ instead of R^2^ to judge the accuracy of the model. In other words, adding new variables, regardless of their usefulness, will always increase the R^2^ value, but only relevant variables will be reflected in the adjusted R^2^. If adjusted R^2^ equals 0.7, then 70% of the variation in the output variable is explained by the input variables [16].

Multiple linear regression analysis revealed that: 1) when CSF Neudesin increases by 1 ng/mL, serum Neudesin concentration increases 1.85 times; 2) with the increase of Neudesin Quotient by 1.0, the serum Neudesin concentration decreases 2.2 times, if the other parameters of the model are fixed (Table 7).

### Univariate and multivariate linear regression analysis results for CSF Neudesin

For CSF Neudesin we did not obtain statistically significant linear regression analysis results, neither univariate nor multivariate.

### Univariate and multivariate linear regression analysis results for Neudesin quotient

Univariate linear regression analysis results for Neudesin Quotient revealed that: 1) with the growth of CSF Neudesin by 1 ng/mL, the value of the Neudesin Quotient increases 1.79 times; 2) with the growth of IL-8 Quotient by 1.0, the value of Neudesin Quotient increases by 1%; 3) for non-tumoral patients the value of Neudesin Quotient decreases 1.6 times compared to patients with an astrocytic brain tumor (Table 8).

**Table 8:** Univariate and multivariate linear regression analysis results for logarithm of Neudesin Quotient

No	Covariate	β	e^β^ (95% CI)	*P*-value
		Univariate linear regression analysis		
1	CSF Neudesin [ng/mL]	0.58	1.79 (1.41–2.26)	< 0.001
2	IL-8 Quotient	0.01	1.01 (1.00–1.02)	0.046
3	Astrocytic brain tumor	−0.47	0.63 (0.40–0.98)	0.041
		Multivariate linear regression analysis		
1	CSF Neudesin [ng/mL]	0.0006	1.0006 (1.0002–1.0009)	0.003
2	CSF IL-8 [pg/mL]	0.59	1.81 (1.48–2.22)	< 0.001

*CI* Confidence Interval, *CSF* cerebrospinal fluid, *IL-8* interleukin 8

In the model of multivariate linear regression analysis, predictor variables influencing Neudesin Quotient included: CSF Neudesin and IL-8 concentration. Adjusted R^2^ for the created model equals 0.59, which indicates that this model explains 59% of the variance in the dependent variable. Multiple linear regression analysis revealed that: 1) when Neudesin concentration increases in CSF by 1 ng/mL, the value of the Neudesin Quotient increases by 1.00; 2) with an increase of CSF IL-8 concentration by 1 pg/mL, the Neudesin Quotient value increases 1.8 times, if the other model parameters are fixed (Table 8).

### Kaplan-Meier survival analysis

Survival status for studied patients was updated in April 2018. The median follow-up was 581 days (range 3–1007 days). During this period 14 (70%) astrocytic brain tumors patients and 2 (25%) meningeal brain tumors patients had died.

Kaplan-Meier survival analysis was performed twice: for total brain tumors group (astrocytic patients plus meningeal patients) and for astrocytic brain tumors group. Due to a small number of meningeal tumors patients, we did not perform Kaplan-Meier survival analysis for this group. Based on Neudesin median concentration, patients were divided into those with Neudesin^Low^ (< median) and those with Neudesin^High^ (≥ median).

**Fig. 1:**
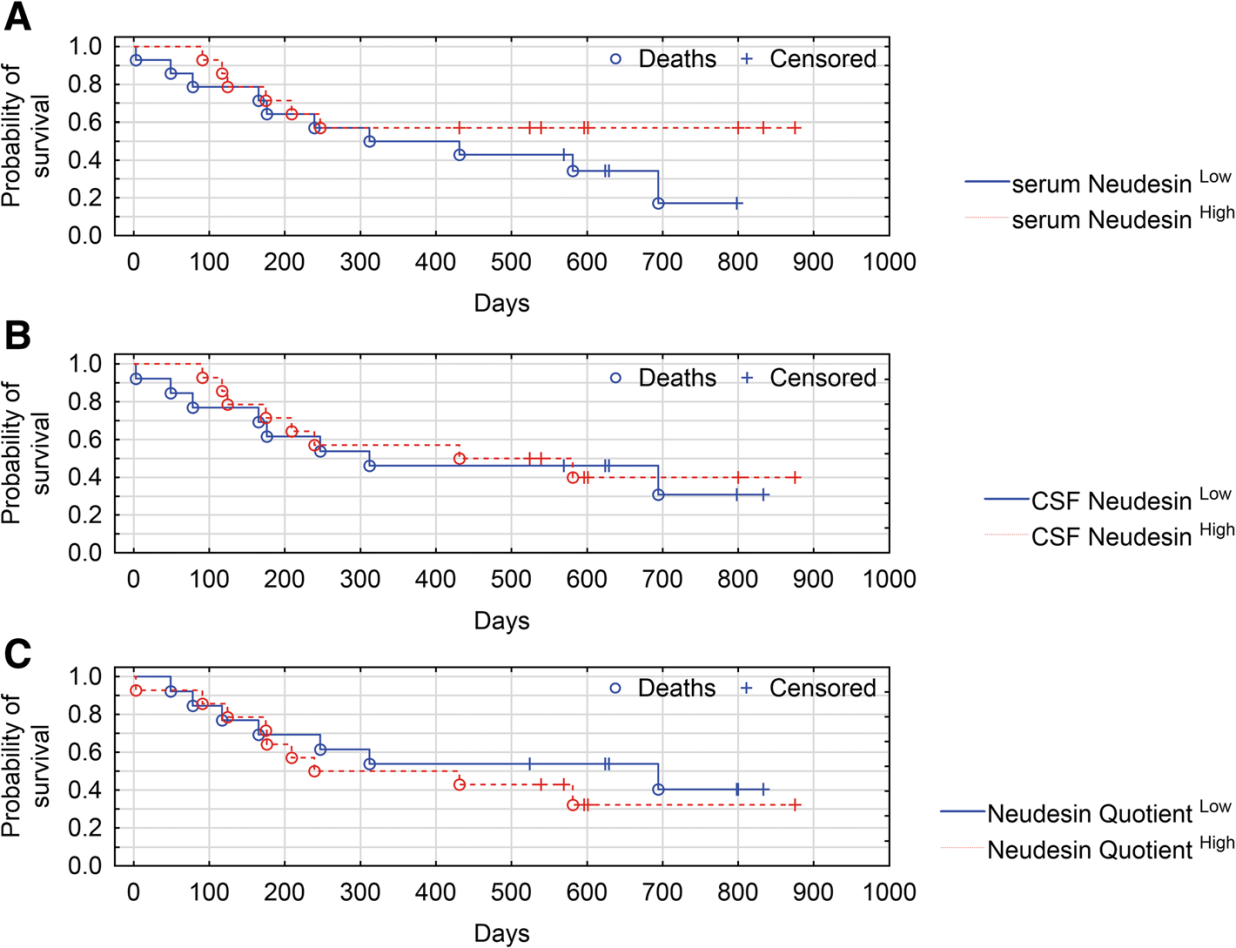
**a**-**c** Kaplan-Meier survival analysis for astrocytic and meningeal brain tumors patients. **a** Patients were divided into serum Neudesin^Low^ and serum Neudesin^High^ subgroups by using a Neudesin cut-off (=median) value of 1.16 ng/mL. **b** Patients were divided into CSF Neudesin^Low^ and CSF Neudesin^High^ subgroups by using a Neudesin cut-off (=median) value of 1.18 ng/mL. **c** Patients were divided into Neudesin Quotient^Low^ and Neudesin Quotient^High^ subgroups by using a Neudesin cut-off (=median) value of 0.98

Based on Neudesin median serum concentration, total brain tumors patients were divided into those with Neudesin < 1.16 ng/mL and those with Neudesin ≥1.16 ng/mL. Based on Neudesin median CSF concentration patients, were divided into those with Neudesin ≤1.18 ng/mL and those with Neudesin ≥1.18 ng/mL. Based on median Neudesin Quotient, patients were divided into those with Neudesin Quotient ≤0.98 and those with Neudesin Quotient ≥0.98. The overall survival (OS) curves of these three total brain tumors Neudesin groups did not significantly differ (*P* = 0.225, *P* = 0.799, and *P* = 0.517, respectively) (Fig. 1a-c).

**Fig. 2:**
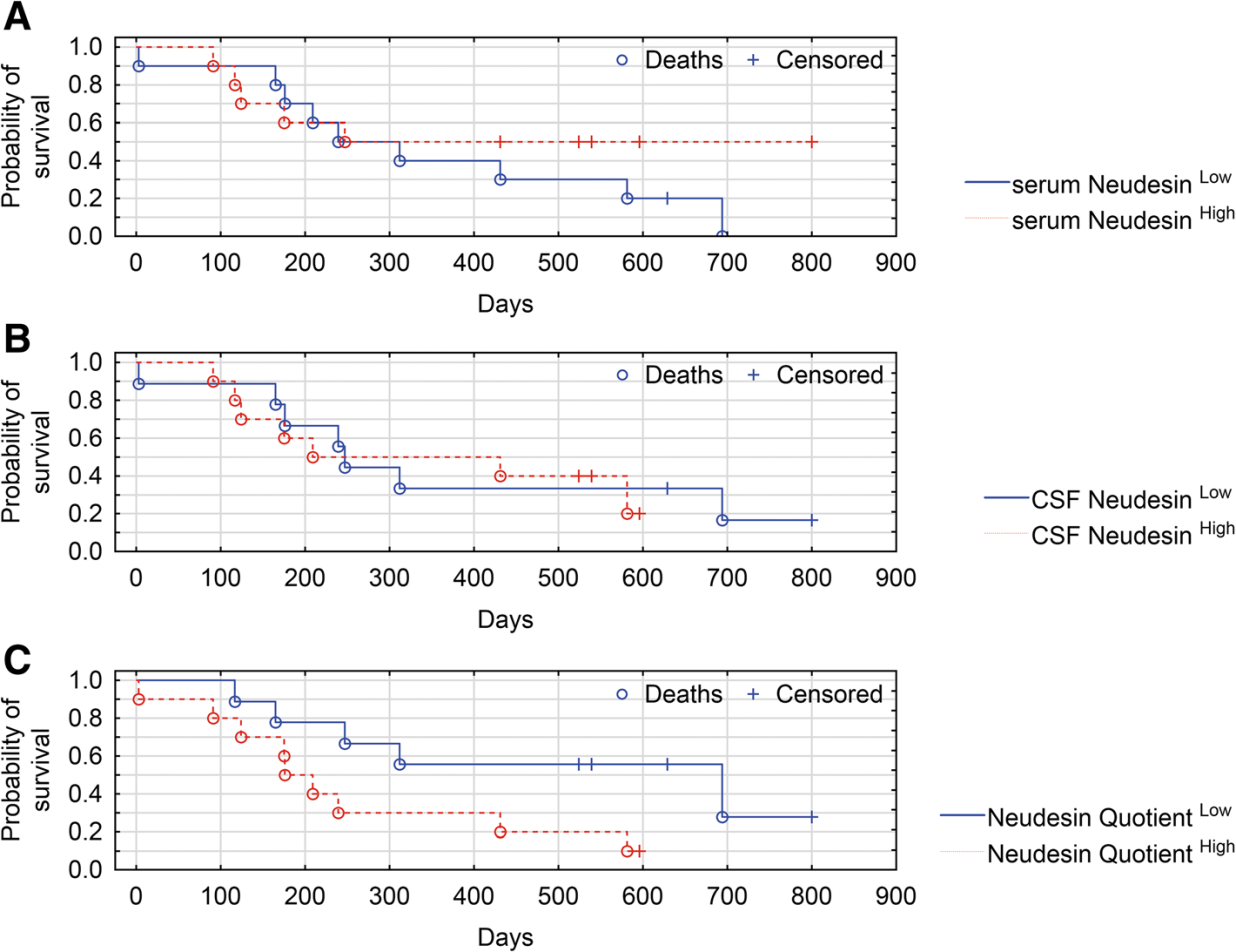
**a**-**c** Kaplan-Meier survival analysis for astrocytic brain tumors patients. **a** Patients were divided into serum Neudesin^Low^ and serum Neudesin^High^ subgroups by using a Neudesin cut-off (=median) value of 1.24 ng/mL. **b** Patients were divided into CSF Neudesin^Low^ and CSF Neudesin^High^ subgroups by using a Neudesin cut-off (=median) value of 1.31 ng/mL. **c** Patients were divided into Neudesin Quotient^Low^ and Neudesin Quotient^High^ subgroups by using a Neudesin cut-off (=median) value of 0.99

Based on Neudesin median serum concentration, astrocytic brain tumors patients were divided into those with Neudesin < 1.24 ng/mL and those with Neudesin ≥1.24 ng/mL. Based on Neudesin median CSF concentration, patients were divided into those with Neudesin < 1.31 ng/mL and those with Neudesin ≥1.31 ng/mL. Based on median Neudesin Quotient, patients were divided into those with Neudesin Quotient < 0.99 and those with Neudesin Quotient ≥0.99. The OS curves of these three astrocytic brain tumors Neudesin groups did not significantly differ (*P* = 0.322, *P* = 0.813, and *P* = 0.060, respectively) (Fig. 2a-c).

## Discussion

The issue of diagnostic and therapeutic response biomarkers of malignant primary brain tumors is still far from solved. Currently, even the use of sophisticated neurosurgical interventions and the latest methods of contemporary oncology does not extend patients’ survival beyond 15 months [17, 18]. In that context, our study identifies a novel circulating biomarker of brain tumors that may be beneficial for their early detection or/and monitoring. To our knowledge, this is the first study to evaluate CSF and serum Neudesin concentrations in patients with a brain tumor as compared to non-tumoral individuals.

We found that non-tumoral patients had statistically lower CSF Neudesin concentrations compared to its serum levels observed in the same group. A similar result was obtained for the meningeal tumor group, however the difference was not significant. On the other hand, CSF Neudesin concentration in the astrocytic brain tumor group showed a tendency to be higher compared to protein concentration in serum, indicative of an increased expression of Neudesin within the central nervous system. This finding is in line with previous studies pointing to high Neudesin expression in several other types of cancer [8, 10].

Closer inspection of the CSF material showed that astrocytic brain tumor patients had statistically higher Neudesin concentrations compared to non-tumoral subjects. Moreover, the median CSF Neudesin value in the astrocytic brain tumor subgroup was nearly 2-fold higher compared to the tumors of the meninges subgroup, however this difference did not register as statistically relevant.

As mentioned above, increased levels of Neudesin were previously found in a number of malignancies [8, 10], and studies suggested its role in the regulation of MAPK and/or AKT pathways crucial for oncogenic signal transduction [4, 7, 19]. Kimura et al. found that under physiological conditions Neudesin is expressed in neuronal, but not glial cells [7], and promotes differentiation of neurons while inhibiting astrocytes’ differentiation [20]. Our results suggest that the development of primary brain tumors may significantly deregulate or alter these mechanisms. Specifically, increased CSF Neudesin concentrations in astrocytic brain tumors compared to the non-tumoral group, may indicate a role of the above mentioned protein in the development of this malignancy. Therefore, increased CSF Neudesin concentrations in astrocytic tumors may be utilized as a circulating biomarker of this disease. Previously we showed increased cerebrospinal fluid IL-8 and decreased Nogo-A concentrations as potential quantitative markers of astrocytic brain tumor presence [11, 12]. Altogether, our previous and current findings identify a potential biomarker panel that may assist in solving the problem of astrocytic brain tumor diagnosis and monitoring. Thus, our study adds to the ongoing effort to identify circulating diagnostic, prognostic, predictive, and therapeutic response biomarkers paramount for improving medical care of the ever-increasing number of patients with gliomas, usually detected after they deeply infiltrate the brain [21].

In our study brain tumor patients (composed of the astrocytic and the meningeal subgroups) had statistically lower serum Neudesin concentrations compared to non-tumoral individuals. Our data indicates that particularly low levels of Neudesin found in the meningeal tumor subgroup highly contributed to the overall differences observed. The reason behind such low levels of Neudesin in meningioma patients is unclear. The molecular machinery of meningiomas, which are the most common intracranial tumors in adults, has still not been fully understood [22]. The available literature on circulating meningioma biomarkers is extremely scarce [11, 22–26]. Interestingly, the histopathological grade of these tumors does not always correlate with their progression/recurrence [22], therefore our findings may help in the understanding of meningioma biology.

Correlation of Neudesin concentration with the previously tested proteins: IL-8, CCL2, sICAM-1, Nogo-A [11, 12] showed a strong positive relationship between serum Neudesin concentrations and CSF Nogo-A levels in the meningeal tumor subgroup. Although the primary role of Nogo-A is to prevent axonal regrowth and sprouting [27], it has been identified as a good marker of primary brain tumors, as it is not expressed in metastatic lesions [28]. Significantly lower CSF Nogo-A concentration in meningeal patients compared to non-tumoral individuals, as reported in our recent manuscript [11], together with the current study, add to our repertoire of biomarkers important for the development of these tumors.

Han et al. [8] revealed that Neudesin increases tumorigenicity and the invasiveness of MCF-7 breast cancer cells. A further study, performed by Stefanska et al. [10], found that silencing of Neudesin decreases cell growth and the invasive abilities of human liver cancer cell lines, and that depletion of Neudesin with selective siRNA reduces human subcutaneous xenograft growth in mice. These studies point to Neudesin as a potential therapeutic target and treatment response biomarker. In order to facilitate such translational endeavors, we subsequently aimed to establish potential factors (e.g.: age, sex, white blood cell count, eGFR value, IL-8, CCL2, sICAM-1, Nogo-A) that may influence the circulating concentration of our protein of interest.

We found that serum Neudesin concentration is influenced by a variety of factors, including a patient’s sex. Univariate linear regression analysis revealed that for women, serum Neudesin concentration was 1.53 times higher than for men. In the previous study we found that the concentrations of Nogo-A – another potential biomarker of primary brain tumors – also depended on a patient’s sex, as women had higher levels of the above-mentioned protein compared to men [11]. Other factors significantly influencing serum Neudesin concentrations are CSF Neudesin, IL-8, and CCL2 levels, white blood cell count and Na + concentrations. Interestingly, in the model of multivariate linear regression analysis, predictor variables influencing serum Neudesin concentrations included only CSF Neudesin concentration and Neudesin Quotient. It should be noted, that the multivariate linear regression model developed in our study explains 82% of the variance in serum Neudesin concentration.

We did not succeed in obtaining statistically significant linear regression models, neither univariate nor multivariate, for CSF Neudesin concentrations. However in the astrocytic brain tumor group CSF Neudesin concentration positively correlated with Neudesin Quotient, the median value of which was statistically higher compared to non-tumoral subjects. Moreover for non-tumoral patients the value of Neudesin Quotient was 1.6 times lower compared to patients with an astrocytic brain tumor.

Both linear regression models, univariate and multivariate, pointed to fewer factors potentially influencing Neudesin Quotient compared to serum Neudesin concentration. This result may indicate that the Neudesin Quotient is a better biomarker of astrocytic brain tumors than Neudesin concentration alone. Our previous studies also reported that for some proteins the calculation of its Quotient (the CSF concentration divided by the serum concentration) revealed higher clinical usefulness than the measurement of this protein in single material, only CSF or blood. For example the Quotient value of chemokine CXCL9 was a better indicator of symptoms resolution in tick-borne encephalitis patients than CSF or serum CXCL9 concentration [29].

The limitation of the present study is a lack of evaluation of Neudesin expression in a larger, available data-set in order to show if the RNA and protein exhibit the same trends, taking into consideration that no report on this is found in the available literature. In truth, we have designed such a study and are currently in the process of applying for funds. We plan to broaden the currently conducted research topic with the study of “molecular parameters”: slowly circulating cellular DNA (coding sequence of Neudesin, Nogo-A, IL-8, CCL2, and sICAM-1 genes) in cerebrospinal fluid/serum, mRNA level for the above proteins in peripheral blood cells (granulocytes/PBMC) and tissues of brain tumors. We also would like to investigate whether changes in expression of the above-mentioned proteins will affect migration, apoptosis and the cell cycle of glioblastoma cells in vitro. We plan to carry out research on commercial cell cultures and glioblastoma cells isolated from patients, which is a step towards so-called “*personalized medicine*”. Nevertheless, we do not think it is good to wait indefinitely for these “full” results and to not endeavor to publish our necessarily preliminary results.

## Conclusion

To conclude, we showed that in astrocytic brain tumor patients Neudesin CSF concentrations are higher compared to non-tumoral individuals and strongly correlate with its serum levels. The factors that correlate with Neudesin levels are very diverse and vary among the groups under analysis (astrocytic brain tumor, meningeal tumor, non-tumoral group). In the cohort of astrocytic brain tumor patients, serum Neudesin concentrations strongly correlate with its CSF levels. Moreover in primary brain tumor patients, serum Neudesin concentrations are clearly gender-dependent. Finally, linear regression models pointed to fewer factors with the potential to influence Neudesin Quotient value, which suggests it as a better biomarker of astrocytic brain tumors than serum and CSF Neudesin concentrations alone.

## Abbreviations

BBB: Blood brain barrier
CNS: Central nervous system
CSF: Cerebrospinal fluid
eGFR: estimated glomerular filtration rate
ELISA: Enzyme-linked immunosorbent assay
ICAM-1/CD54: Intercellular adhesion molecule-1
IL-8/CXCL8: Interleukin-8
K^+^: Potassium
MAP: Mitogen-activated protein
MAPR: Membrane-associated progesterone receptor
MCP-1/CCL2: Monocyte chemotactic protein-1
Na^+^: Sodium
Nogo-A: Neurite growth isoform A of reticulon-4
PI3K: Phosphatidylinositol 3-kinase protein
RNA: Messenger ribonucleic acid
WBC: White blood cells count
WHO: World Health Organization
